# Protein expression in female salivary glands of pyrethroid-susceptible and resistant strains of *Aedes aegypti* mosquitoes

**DOI:** 10.1186/s13071-019-3374-2

**Published:** 2019-03-14

**Authors:** Chonlada Mano, Narissara Jariyapan, Sriwatapron Sor-Suwan, Sittiruk Roytrakul, Suthathip Kittisenachai, Pongsri Tippawangkosol, Pradya Somboon

**Affiliations:** 1 Faculty of Medicine, Chiang Mai University, Chiang Mai, 50200 Thailand; 2 Center of Insect Vector Study, Department of Parasitology, Faculty of Medicine, Chiang Mai University, Chiang Mai, Thailand; 3 Department of Parasitology, Faculty of Medicine, Chulalongkorn University, Bangkok, Thailand; 4 National Center for Genetic Engineering and Biotechnology (BIOTEC), National Science and Technology Development Agency, Pathumthani, Thailand

**Keywords:** *Aedes aegypti*, Insecticide resistance, Pyrethroids, Salivary glands, Mosquito salivary proteins

## Abstract

**Background:**

A group of insecticides called pyrethroids has been used extensively worldwide and development of pyrethroid resistance within mosquito populations, especially in *Aedes aegypti*, has rapidly spread through populations. In this study, SDS-PAGE, 2-DE coupled with NanoLC-MS, and bioinformatics were used to analyze the female salivary gland proteins of pyrethroid-susceptible (PMD) and pyrethroid-resistant (PMD-R and UPK-R) strains of *Ae. aegypti* mosquitoes for the first time.

**Results:**

SDS-PAGE analysis revealed that among the three strains at least nine major proteins were detected but one protein band (20 kDa) was found only in the PMD strain. Two-dimensional gel electrophoresis analysis revealed 19 similarly expressed proteins in the salivary glands of the three strains involved in blood-feeding process, stress response, immunogenic response, and metabolic process and five additional major protein spots differentially expressed in the susceptible and resistant strains. Comparative analysis of the expression volume of each protein spot between the PMD and the PMD-R strains showed three downregulated proteins of the PMD-R mosquitoes. For UPK-R strains, six major proteins were downregulated when compared to the PMD strain. Additionally, four downregulated proteins were found in the UPK-R when compared to the PMD-R strain. These results suggest that pyrethroids might induce alteration of salivary gland proteins in resistant mosquitoes. Network analysis by STITCH database 5.0 showed that SRPN23 interacted with sodium and calcium ions, suggesting that SRPN23 might be involved in insecticide resistance.

**Conclusions:**

Information obtained from this study will be useful for further studies on the roles of differentially expressed salivary gland proteins in resistance to insecticides and viral transmission.

**Electronic supplementary material:**

The online version of this article (10.1186/s13071-019-3374-2) contains supplementary material, which is available to authorized users.

## Background

*Aedes aegypti* mosquitoes serve as the primary vector of dengue hemorrhagic fever (DHF), yellow fever, chikungunya fever and Zika fever. The diseases are considered as serious public health problems in several countries in tropical and subtropical areas. The diseases cause mortality and morbidity of populations; however, there is a lack of vaccines or specific treatments. Therefore, mosquito control is an essential method to control the transmission of the diseases. One of the popular methods used to reduce the population of mosquitoes is the application of chemical compounds such as insecticides. However, this method is impeded by the development of resistance within mosquito populations [1, 2].

In Thailand, at least four groups of synthetic compounds, organochlorine (DDT), organophosphates (temephos, fenitrothion, malathion and chlorpyrifos), carbamates (propoxur, pirimiphosmethyl and bendiocarb) and pyrethroids (permethrin, deltamethrin, lambda-cyhalothrin and etofenprox) have been extensively used to control mosquito vectors. At present, several mosquitoes have been reported as resistant to insecticides, especially to DDT, pyrethroids (i.e. permethrin and deltamethrin), carbamates (i.e. propoxur) and organophosphates (i.e., temephos and fenitrothion) [3, 4].

The insecticide susceptible Pang Mei Daeng (PMD) and Pang Mei Daeng resistant (PMD-R) strains of *Ae. aegypti* from Chiang Mai, Thailand, have been used in several previous studies [5–9]. The PMD strain is susceptible to pyrethroids, but resistant to DDT, which has mainly been attributed to increased DDTase activity [5]. No *kdr* mutations (S989P, V1016G and F1534C) have been found in this strain [10]. PMD-R (S/S989 + V/V1016 + C/C1534, or SS + VV + CC) is homozygous for C1534, lacks S989P and V1016G *kdr* mutations, and is resistant to both DDT and permethrin but susceptible to deltamethrin [10–12]. Recently, the Upakhut resistant (UPK-R) strain has also been established from mosquitoes collected from Wat Upakhut in the city of Chiang Mai. The UPK-R strain is homozygous for the G1016 *kdr* allele and resistant to DDT, permethrin and deltamethrin. It harbors P/P989 + G/G1016 + F/F1534, or PP + GG + FF [13–15].

An important role of the mosquito salivary glands is to modulate host responses that facilitate transmission of pathogens. Dengue virus is transmitted to a vertebrate host while saliva of infected female mosquitoes is injected into the host. A recent study demonstrated that *Ae. aegypti* salivary gland extract enhances dengue pathogenesis after infection [16]. In previous studies, differential expression of salivary gland proteins between insecticide-susceptible and resistant strains of *Culex quinquefasciatus* [17], *Anopheles gambiae* [18] and *Anopheles stephensi* [19] has been reported. Regarding insecticide resistant mosquitoes in Thailand, no report of differentially expressed salivary gland proteins between susceptible and insecticide-resistant *Ae. aegypti* mosquitoes is available.

In the present study, SDS-PAGE, two-dimensional gel electrophoresis (2-DE) coupled with mass-spectrometry (NanoLC-MS), and bioinformatics were used to compare salivary gland expression profiles and identify the differentially expressed proteins in the three *Ae. aegypti* strains, PMD, PMD-R and UPK-R. The information on proteins in the salivary glands of these insecticide-resistant mosquitoes might help to explain their impact on vectorial capacity.

## Results

### Insecticide susceptibility test

To confirm the insecticide susceptibility status of the *Ae. aegypti* PMD, PMD-R, and UPK-R strains, the mosquito strains were tested according to the WHO standard method [20]. The results revealed that the PMD strain was susceptible to both permethrin and deltamethrin, while the PMD-R strain was resistant to permethrin with 13.33% mortality and the UPK-R strain was resistant to permethrin and deltamethrin with zero and 1.67% mortality, respectively (Table 1).

**Table 1:** Insecticide susceptibility status of *Ae. aegypti* PMD, PMD-R and UPK-R strains

Strain	Insecticide	Total no. of tested mosquitoes	Total no. of mosquito deaths	Mortality rate (%)	Status^a^
PMD	Permethrin	300	300	100	S
	Deltamethrin	300	300	100	S
PMD-R	Permethrin	300	40	13.33	R
	Deltamethrin	300	300	100	S
UPK-R	Permethrin	300	0	0	R
	Deltamethrin	300	5	1.67	R

^a^Interpretation of mortality rate (WHO, 2013)

*Abbreviations*: R, resistance; S, susceptibility

### Comparison of female salivary gland protein profiles of *Ae. aegypti* mosquitoes between susceptible and resistant strains by SDS-PAGE

**Fig. 1:**
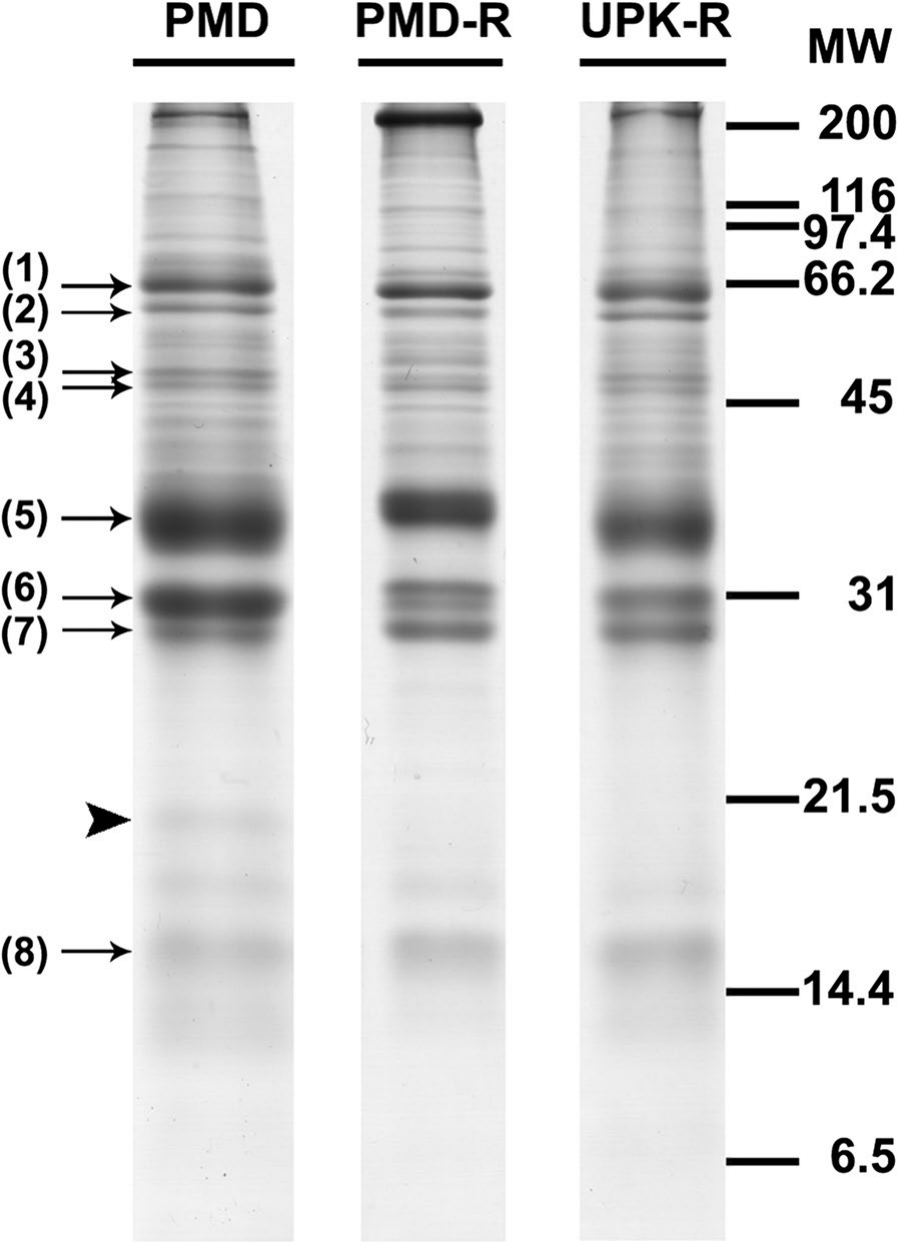
Electrophoretic protein profiles of salivary glands obtained from female *Ae. aegypti* mosquitoes of PMD, PMD-R, and UPK-R strains. Proteins of five pairs of salivary glands were separated on a 15% SDS polyacrylamide gel and stained with CBB. Molecular mass markers are indicated on the right in kDa. Arrows indicate major protein bands similarly expressed among the three strains. The arrowhead indicates a protein band observed only in the PMD strain

A comparative analysis of the female salivary gland protein profiles among the PMD, PMD-R and UPK-R strains was performed using SDS-PAGE. The SDS-PAGE results showed that nine major proteins were detected in the three strains (Fig. 1). The molecular weights of these protein bands were estimated to be 66, 60, 49, 47, 37, 31, 29, 18 and 16 kilodaltons (kDa), respectively. However, one protein band (20 kDa) was found only in the PMD strain. As a protein band may consist of more than one protein, 2-DE was therefore used for further detailed analysis.

### Comparison of female salivary gland protein profiles of *Ae. aegypti* mosquitoes between susceptible and resistant strains by 2-DE

**Fig. 2:**
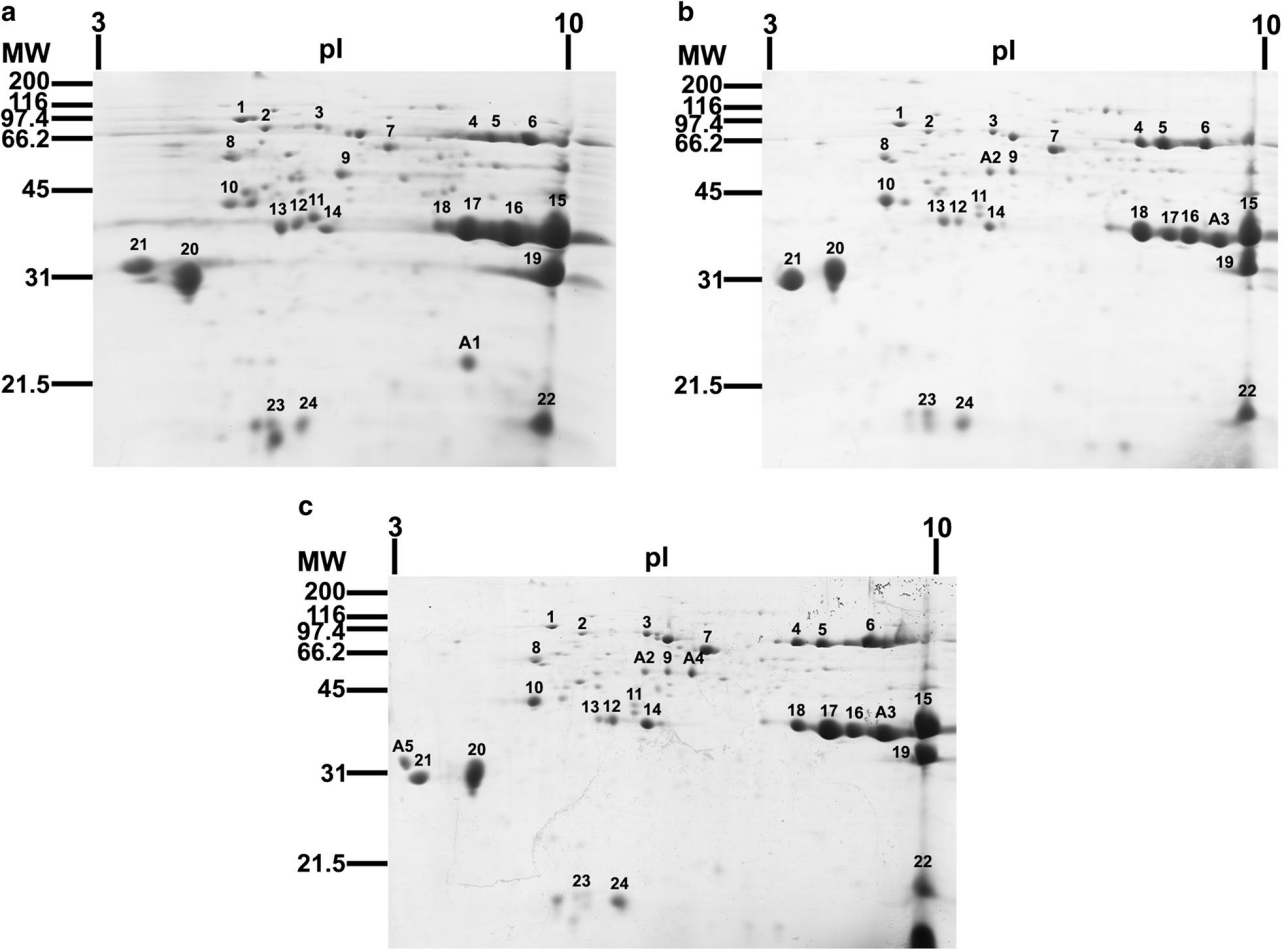
Comparison of representative 2-DE protein profiles from 60 female salivary glands of *Ae. aegypti* mosquitoes. **a** PMD, **b** PMD-R and **c** UPK-R strains. Proteins were separated in the first dimension by IEF using a 7 cm strip, pH 3–10. Separation in the second dimension was performed using 15% SDS-PAGE followed by CBB staining. Molecular mass markers are indicated on the left in kDa. Isoelectric points (pI) are indicated at the top. Numbers indicate major spots found in all three strains. Letters (A1-A5) indicate major spots found specifically in each strain

Protein profiles of the female mosquitoes of the PMD, PMD-R and UPK-R strains were also analyzed by 2-DE (Fig. 2). Two-dimensional gel electrophoresis (2-DE) was performed on three biological replicates and images (Additional file 1: Figure S1) were analyzed using Image Master^TM^ Platinum 7.0 software (GE Healthcare, Buckinghamshire, UK). The molecular weight (MW), isoelectric point (pI) and normalized volume of each protein spot from each independent gel image were calculated following instructions for the software supplied by the manufacturer. Then, the average normalized volume (ANV) and standard deviation (SD) of the same protein (same MW and pI) from the three replicated 2-DE gel images were also calculated by the software. In this study, 57, 45 and 52 protein spots were detected in the PMD, PMD-R and UPK-R strains, respectively. Protein spots with ANV ≥ 0.1 were selected as major proteins. Results showed that at least 24 major protein spots were detected in each strain. The molecular weight of these proteins varied between 15 and 75 kDa, with pI ranging between 3.5 and 9.5. It was noted that the A1 spot was specific to the PMD strain (Fig. 2a) whereas the A2 and A3 spots were found in both the PMD-R (Fig. 2b) and UPK-R (Fig. 2c) strains. The A4 and A5 spots were found only in the UPK-R strain.

### Identification of female salivary gland proteins of *Ae. aegypti* PMD, PMD-R and UPK-R strains by NanoLC-MS analysis

In order to identify the detected salivary gland proteins, a total of 154 protein spots comprised of 57, 45 and 52 protein spots from the PMD, PMD-R and UPK-R strains, respectively, were subjected to NanoLC-MS analysis. For the major protein spots, the relevant spot from two independent gels was used for NanoLC-MS analysis to confirm the identification. The results reported here refer to the identification by Mascot against the nonredundant NCBInr_Metazoa database. Of these, 24 major protein spots were identified as similar proteins in all strains and only the best hit of the proteins from *Ae. aegypti* is reported in Table 2. The mass list (list of peptides) found for each major spot analyzed on the three mosquito strains is shown in Additional file 2: Table S1. These proteins included AAEL017349-PA, which was spot number 1 (SN1), heat-shock cognate 70 (SN2), putative secreted protein (SN3), apyrase (SN4 and 5), AAEL006333-PA/5’-nucleotidase (SN6), AAEL005672-PA/putative adenosine deaminase (SN7), AAEL000641-PA (SN8), salivary anti-FXa serpin (SN9), putative purine hydrolase (SN10), angiopoietin-like protein (SN11), putative 34 kDa secreted protein (SN12 and 13), putative 34 kDa family secreted salivary protein (SN14), D7 protein family (SN15, 16, 17 and 18), AAEL000793-PA/putative secreted protein (SN19), AAEL010228-PA/putative 19.6 kDa secreted protein (SN20), 30 kDa salivary gland allergen variant 3 (SN21), short form D7Cclu23 salivary protein (SN22), putative C-type lectin/AAEL000533-PA (SN23) and short salivary D7/putative 16.9 kDa secreted protein (SN24). For the differentially expressed protein spots, the A1, A2 and A4 spots were identified as a short D7 protein, a salivary anti-FXa serpin and a salivary serpin, respectively (Table 2). Unfortunately, the A3 and A5 spots could not be identified in our study.

**Table 2:** A list of identified female salivary gland proteins of *Ae. aegypti* PMD, PMD-R, and UPK-R strains by NanoLC-MS analysis

SN^a^ (MW/pI)	Strain	Accession number^b^	Protein description	Prot. score^c^	PS^d^	No. P/ %C^e^	Database MW/pI	Peptide sequence(s)	Gene name^f^
1 (75/5.3)	PMD	XP_011493320	AAEL017349-PA	65	36	2/3	72.4/5.1	R.LTPEDIER.M	AAEL017349-PA
					29			K.VADDDKATMEEAIDEK.I + Oxidation (M)	
	PMD-R	XP_011493320	AAEL017349-PA	65	36	2/3	72.4/5.1	R.LTPEDIER.M	AAEL017349-PA
					29			K.VADDDKATMEEAIDEK.I + Oxidation (M)	
	UPK-R	XP_011493320	AAEL017349-PA	271	66	4/7	72.4/5.1	K.IVITNDQNR.L	AAEL017349-PA
					71			K.FEELNMDLFR.S + Oxidation (M)	
					63			K.DVDEIVLVGGSTR.I	
					75			K.VFAPEEISAMVLGK.M + Oxidation (M)	
2 (73/5.6)	PMD	ABF18332	Heat-shock cognate 70	40	40	1/1	71.4/5.3	K.VEIIANDQGNR.T	na^g^
	PMD-R	ABF18332	Heat-shock cognate 70	49	49	1/1	71.4/5.3	K.VEIIANDQGNR.T	na
	UPK-R	ABF18332	Heat-shock cognate 70	163	80	2/3	71.4/5.3	K.DAGTISGLNVLR.I	na
					83			R.FEELNADLFR.S	
3 (72/6.2)	PMD	AAL76020	Putative secreted protein	84	84	1/1	66.6/5.8	K.TASNDCTPFR.L	AAEL000748
	PMD-R	AAL76020	Putative secreted protein	84	84	1/1	66.6/5.8	K.TASNDCTPFR.L	AAEL000748
	UPK-R	AAL76020	Putative secreted protein	130	62	2/4	66.6/5.8	K.DLEGITDELGK.V	AAEL000748
					67			K.DIATLEQNVNTLHK.D	
4 (69/8.5)	PMD	AAC37218	Apyrase	231	65	3/6	63.2/8.5	R.TGPLDSDVFK.N	APY
					82			K.VTLSNAVEAVR.R	
					84			K.VEAIGSTVVGETK.I	
	PMD-R	AAC37218	Apyrase	449	66	6/12	63.2/8.5	R.TGPLDSDVFK.N	APY
					61			R.TNCLQVSGLR.I	
					76			K.VTLSNAVEAVR.R	
					73			K.VEAIGSTVVGETK.I	
					96			K.KIEVMDYTNPK.S + Oxidation (M)	
					80			K.DKVEGPYPTIVESK.N	
	UPK-R	AAC37218	Apyrase	231	81	3/5	63.2/8.5	K.IGIIGVLYDK.T	APY
					72			R.IVIDISKPIR.S	
					78			K.VTLSNAVEAVR.R	
5 (68/8.9)	PMD	AAC37218	Apyrase	137	59	2/4	63.2/8.5	R.TNCLQVSGLR.I	APY
					75			K.VEAIGSTVVGETK.I	
	PMD-R	AAC37218	Apyrase	531	66	7/12	63.2/8.5	R.TGPLDSDVFK.N	APY
					71			K.IGIIGVLYDK.T	
					69			R.TNCLQVSGLR.I	
					87			K.VTLSNAVEAVR.R	
					68			R.RTGPLDSDVFK.N	
					79			K.VEAIGSTVVGETK.I	
					93			K.DKVEGPYPTIVESK.N	
	UPK-R	AAC37218	Apyrase	641	61	9/17	63.2/8.5	R.TGPLDSDVFK.N	APY
					81			K.IGIIGVLYDK.T	
					64			R.TNCLQVSGLR.I	
					70			R.IVIDISKPIR.S	
					79			K.VTLSNAVEAVR.R	
					59			K.GLAPYLAELNK.E	
					60			K.VEAIGSTVVGETK.I	
					80			K.KIEVMDYTNPK.S + Oxidation (M)	
					63			K.DKVEGPYPTIVESK.N	
6 (68/9.5)	PMD	XP_001651903	AAEL006333-PA	139	80	2/3	63.9/8.7	R.ISLAAGAITR.G	AAEL006333
					61			K.LELSNDCR.L	
	PMD-R	XP_001651903	AAEL006333-PA	139	80	2/3	63.9/8.7	R.ISLAAGAITR.G	AAEL006333
					61			K.LELSNDCR.L	
	UPK-R	ABF18486	5’-nucleotidase	96	20	3/6	61.8/8.9	R.VTVLGSTIK.K	AAEL006333
					38			K.VGESKLELSNDCR.L	
					39			R.GQILTALPFNSNANR.V	
7 (66/7.2)	PMD	XP_001651237	AAEL005672-PA	593	37	11/23	59.9/6.4	R.LKLEEMVK.G + Oxidation (M)	AAEL005672
					48			R.HIFEVLDR.F	
					45			R.FATDDEFLK.I	
					35			K.SNHPEFIGAK.F	
					51			K.EWSLVSEIR.Q	
					51			R.SLLEFAPALLK.L	
					52			K.GDFGVSHGPQFK.C	
					70			R.GVLPDVYDLDGK.I	
					87			K.TDQNLIDAVLLGSK.R	
					62			K.QFYDDHVQYLEFR.G	
					54			K.QLALNSIEYSAMNSEEK.T + Oxidation (M)	
	PMD-R	AAL76033	Putative adenosine deaminase	100	22	3/4	60.6/6.5	K.FIYAPGR.F	AAEL005672
					36			K.SSHPEFIGAK.F	
					45			R.FATDDEFLK.I	
	UPK-R	AAL76033	Putative adenosine deaminase	219	70	3/7	60.6/6.5	R.GVLPDVYDLDGK.I	AAEL005672
					87			K.TDQNLIDAVLLGSK.R	
					62			K.QFYDDHVQYLEFR.G	
8 (62/5.1)	PMD	XP_001649774	AAEL000641-PA	163	77	2/4	56.3/5.0	K.MDATANELEHTK.I + Oxidation (M)	AAEL000641
					86			K.VDATEEQELSEK.H	
	PMD-R	XP_001649774	AAEL000641-PA	47	47	1/2	56.3/5.0	R.NGTPIEYTGGR.E	AAEL000641
	UPK-R	XP_001649774	AAEL000641-PA	82	82	1/2	56.3/5.0	K.VDATEEQELSEK.H	AAEL000641
9 (59/6.6)	PMD	ABF18028	Salivary anti-FXa serpin	121	59	2/5	48.3/6.1	R.MGQVINDALK.N + Oxidation (M)	SRPN23
					62			K.TPPEAAMGLEDK.Q + Oxidation (M)	
	PMD-R	ABF18028	Salivary anti-FXa serpin	58	58	1/2	48.3/6.1	R.TAMAVAIGSEK.V + Oxidation (M)	SRPN23
	UPK-R	ABF18028	Salivary anti- FXa serpin	307	59	4/11	48.3/6.1	R.TAMAVAIGSEK.V + Oxidation (M)	SRPN23
					77			R.MGQVINDALK.N + Oxidation (M)	
					106			R.IPQFGLQTTVPGR.Q	
					64			K.VFEQGQDVALGEIVQK.M	
10 (49/5.1)	PMD	AAL76010	Putative purine hydrolase	48	48	1/2	38.2/5.0	R.QMVEDLNR.- + Oxidation (M)	AAEL006485
	PMD-R	AAL76010	Putative purine hydrolase	76	42	2/4	38.2/5.0	R.LITPAPR.R	AAEL006485
					34			R.QMVEDLNR.- + Oxidation (M)	
	UPK-R	AAL76010	Putative purine hydrolase	73	73	1/3	38.2/5.0	K.SEIAGIYILGGNR.N	AAEL006485
11 (48/6.0)	PMD	ABF18025	Angiopoietin-like protein variant, partial	68	22	2/6	33.5/5.7	K.FVVGPEEQR.Y	AAEL000749
					45			K.FSDVSNTPLK.L	
	PMD-R	AAL76032	Angiopoietin-like protein	68	22	2/6	33.5/5.7	K.FVVGPEEQR.Y	AAEL000749
					45			K.FSDVSNTPLK.L	
	UPK-R	ABF18025	Angiopoietin-like protein variant, partial	153	37	4/15	33.5/5.7	K.FVVGPEEQR.Y	AAEL000749
					32			K.FSDVSNTPLK.L	
					35			K.SSGLTTIPIGSEPR.V	
					49			R.FTQMFSQQFYR.H + Oxidation (M)	
12 (45/5.8)	PMD	ABF18018	Putative 34 kDa secreted protein	64	64	1/3	36.4/5.2	K.LQEEIEEQTK.Q	AAEL003601
	PMD-R	ABF18018	Putative 34 kDa secreted protein	64	64	1/3	36.4/5.2	K.LQEEIEEQTK.Q	AAEL003601
	UPK-R	ABF18018	Putative 34 kDa secreted protein	132	65	2/6	36.4/5.2	K.SVATEIVQMR.D + Oxidation (M)	AAEL003601
					68			K.QNFEDQVNQIVK.S	
13 (46/5.7)	PMD	AAL76018	Putative 34 kDa secreted protein	54	54	1/4	36.4/5.2	R.DSAISTDQVDQLK.Q	AAEL003601
	PMD-R	AAL76018	Putative 34 kDa secreted protein	155	74	2/7	36.4/5.2	R.DSAISTDQVDQLK.Q	AAEL003601
					81			K.QNFEDQVNQIVK.S	
	UPK-R	AAL76018	Putative 34 kDa secreted protein	176	40	4/12	36.4/5.2	R.IYLGALR.F	AAEL003601
					54			K.SVATEIVQMR.D + Oxidation (M)	
					51			K.VTELEQQIAK.Q	
					35			K.QNFEDQVNQIVK.S	
14 (43/6.2)	PMD	ABF18017	Putative 34 kDa family secreted salivary protein	98	49	2/6	36.0/6.1	K.LQEEIEEQTK.Q	AAEL003600
					53			K.QCNLSEDDLTK.L	
	PMD-R	ABF18017	Putative 34 kDa family secreted salivary protein	206	73	3/12	36.0/6.1	K.QCNLSEDDLTK.L	AAEL003600
					75			K.TQGVSNMEVQLLR.E + Oxidation (M)	
					60			R.QANQDTSKAEGEMVEK.I + Oxidation (M)	
	UPK-R	ABF18017	Putative 34 kDa family secreted salivary protein	256	66	3/8	36.0/6.1	K.VLNTILDQVNK.L	AAEL003600
					86			R.KVLNTILDQVNK.L	
					106			K.TQGVSNMEVQLLR.E	
15 (40/9.5)	PMD	AAL16049	Long form D7Bclu1 salivary protein	450	64	6/18	39.2/9.4	K.NELDVDEIAR.D	AAEL006417
					93			R.SKDYFAALTGK.L	
					74			K.SSSCSEVFEAFK.K	
					75			R.KQVDDIDKIQCS.-	
					63			K.SSSCSEVFEAFKK.V	
					84			K.LEPSDDQATQCYTK.C	
	PMD-R	XP_001657778	AAEL006417-PA	614	58	8/29	39.2/9.4	K.IGFYEPGEKR.F	AAEL006417
					91			R.SKDYFAALTGK.L	
					68			K.YQMGSGIVFER.H + Oxidation (M)	
					83			K.SSSCSEVFEAFK.K	
					72			R.KQVDDIDKIQCS.-	
					68			K.VTNDFKEAFDYR.E	
					88			K.LEPSDDQATQCYTK.C	
					88			R.YMTSKNELDVDEIAR.D + Oxidation (M)	
	UPK-R	AAL16049	Long form D7Bclu1 salivary protein	385	63	5/14	39.2/9.4	K.AALQNWLGWK.L	AAEL006417
					82			R.SKDYFAALTGK.L	
					87			K.SSSCSEVFEAFK.K	
					60			K.SSSCSEVFEAFKK.V	
					94			K.LEPSDDQATQCYTK.C	
16 (39/9.5)	PMD	AAA29347	D7 protein	437	72	6/21	37.6/8.4	K.SKVEAYANAVK.Q	D7
					71			K.FDASVIQEQFK.A	
					58			R.QYTVLDDALFK.E	
					75			K.DNQLDVEEVKR.D	
					85			K.CLVESSVKDDFK.E	
					83			R.CMEDNLEDGANR.L + Oxidation (M)	
	PMD-R	AAA29347	D7 protein	363	66	5/17	37.6/8.4	K.SKVEAYANAVK.Q	D7
					60			K.DDFKEAFDYR.E	
					69			K.FDASVIQEQFK.A	
					72			K.QSYFEFCENK.Y	
					102			K.QLPSTNNDCAAVFK.A	
	UPK-R	AAA29347	D7 protein	390	61	6/21	37.6/8.4	R.TGLYDPVAQK.F	D7
					68			K.SKVEAYANAVK.Q	
					71			K.FDASVIQEQFK.A	
					67			R.QYTVLDDALFK.E	
					63			K.DNQLDVEEVKR.D	
					70			K.QLPSTNNDCAAVFK.A	
17 (40/8.9)	PMD	AAA29348	D7 protein	153	55	3/9	37.5/8.5	R.TGLYDPVAQK.F	D7
					75			K.NNELDAEEVKR.D	
					23			K.KSYFEFCENK.Y	
	PMD-R	AAA29348	D7 protein	191	65	3/10	37.5/8.5	K.FDASVIQEQFK.A	D7
					71			R.QYTVLDDALFK.E	
					59			K.NNELDAEEVKR.D	
	UPK-R	AAA29347	D7 protein	223	67	3/10	37.6/8.4	R.QYTVLDDALFK.E	D7
					84			K.DNQLDVEEVKR.D	
					76			K.CLVESSVKDDFK.E	
18 (42/8.9)	PMD	AAA29348	D7 protein	75	75	1/3	37.5/8.5	K.NNELDAEEVKR.D	D7
	PMD-R	AAA29348	D7 protein	208	63	3/9	37.5/8.5	K.DDFKEAFDYR.E	D7
					73			K.FDASVIQEQFK.A	
					74			K.KSYFEFCENK.Y	
	UPK-R	AAA29348	D7 protein	211	74	3/9	37.5/8.5	K.FDASVIQEQFK.A	D7
					67			R.QYTVLDDALFK.E	
					74			K.KSYFEFCENK.Y	
19 (33/9.5)	PMD	XP_001651000	AAEL000793-PA	199	87	2/16	29.6/9.1	R.MPTLVWDDELAHIASFNAR.K + Oxidation (M)	AAEL000793
					112			R.RGPHVACNAPTQFGSACGQEPK.F	
	PMD-R	AAL76012	Putative secreted protein	199	87	2/16	29.6/9.1	R.MPTLVWDDELAHIASFNAR.K + Oxidation (M)	AAEL000793
					112			R.RGPHVACNAPTQFGSACGQEPK.F	
	UPK-R	AAL76009	Putative secreted protein	38	38	1/3	30.2/9.0	K.DYCSSVFR.K	AAEL003053
20 (33/4.6)	PMD	AAX54871	Putative 19.6 kDa secreted protein	31	31	1/4	21.8/4.8	K.VIEQLDQIK.V	AAEL010228
	PMD-R	XP_001660690	AAEL010228-PA	166	62	2/12	21.8/4.8	K.DSNAYQCSQDR.S	AAEL010228
					104			R.NPIVDVIGSAGDFSK.I	
	UPK-R	XP_001660690	AAEL010228-PA	173	66	2/12	21.8/4.8	K.DSNAYQCSQDR.S	AAEL010228
					107			R.NPIVDVIGSAGDFSK.I	
21 (31/4.1)	PMD	ABF18125	30 kDa salivary gland allergen variant 3	124	64	2/6	27.9/4.2	R.VPVVEAIGR.I	AAEL010235
					61			K.NDPADTYR.Q	
	PMD-R	ABF18125	30 kDa salivary gland allergen variant 3	452	64	6/18	27.9/4.2	R.VPVVEAIGR.I	AAEL010235
					61			R.QVVALLDKDTK.V	
					86			R.SALNNDLQSEVR.V	
					75			K.SEYQCSEDSFAAAK.S	
					80			K.KSEYQCSEDSFAAAK.S	
					88			K.KKSEYQCSEDSFAAAK.S	
	UPK-R	ABF18125	30 kDa salivary gland allergen variant 3	301	61	4/17	27.9/4.2	K.NDPADTYR.Q	AAEL010235
					62			R.QVVALLDKDTK.V	
					107			R.SALNNDLQSEVR.V	
					71			K.SEYQCSEDSFAAAK.S	
22 (15/9.5)	PMD	AAL16050	Short form D7Cclu23 salivary protein	32	32	1/6	18.2/9.0	R.FFTEKPFCK.-	AAEL006423
	PMD-R	AAL16050	Short form D7Cclu23 salivary protein	163	47	4/22	18.2/9.1	K.INGWAYFR.C	AAEL006423
					46			R.FFTEKPFCK.-	
					54			R.NFLCHTANFK.L	
					16			K.TEMGDKINGWAYFR.C + Oxidation (M)	
	UPK-R	AAL16050	Short form D7Cclu23 salivary protein	32	32	1/6	18.2/9.1	R.FFTEKPFCK.-	AAEL006423
23 (15/5.6)	PMD	AAL76029	Putative C-type lectin	33	33	1/6	18.1/5.3	K.QQQVEELAQR.V	CTL16
	PMD-R	XP_001647974	AAEL000533-PA	87	46	2/12	18.1/5.3	R.LAVLDSEQK.Q	CTL16
					40			K.QQQVEELAQR.V	
	UPK-R	AAL76029	Putative C-type lectin	93	51	2/12	18.1/5.3	R.LAVLDSEEK.Q	CTL16
					42			K.QQQVEELAQR.V	
24 (15/5.9)	PMD	AAL76035	Putative 16.9 kDa secreted protein	135	23	3/20	17.4/5.5	K.TELDEVTK.E	SG14.5sp
					32			R.NLQVDGNMPK.V + Oxidation (M)	
					80			K.VAQDMVPYGFNIK.T + Oxidation (M)	
	PMD-R	ABF18031	Short salivary D7 protein, partial	80	80	1/8	17.5/5.7	K.VAQDMVPYGFNIK.T + Oxidation (M)	na
	UPK-R	ABF18031	Short salivary D7 protein, partial	75	75	1/8	17.5/5.7	K.VAQDMVPYGFNIK.T + Oxidation (M)	na
A1 (20/8.6)	PMD	ABF18082	Short D7 protein	44	44	1/5	18.6/8.2	R.QNQIEELNK.L	SG16.2sp
A2 (56/6.2)	PMD-R	ABF18028	Salivary anti-FXa serpin	229	70	3/8	48.3/6.1	R.TAMAVAIGSEK.V + Oxidation (M)	SRPN23
					69			K.MEMSIAADGEK.Q + Oxidation (M)	
					90			K.TPPEAAMGLEDK.Q + Oxidation (M)	
	UPK-R	ABF18028	Salivary anti-FXa serpin	95	95	1/3	48.3/6.1	R.IPQFGLQTTVPGR.Q	SRPN23
A4 (59/6.2)	UPK-R	ABF18509	Salivary serpin	235	62	3/8	48.2/6.3	R.MGQVINDALK.N + Oxidation (M)	SRPN23
					68			K.TPPEAGMGLEDK.Q + Oxidation (M)	
					105			R.IPQFGLQTTVPGR.Q	

^a^Spot number refers to those shown in Fig. 2 /(Observed MW/pI)

^b^Accession number of the best hit of proteins from *Ae. aegypti*, available at http://www.ncbi.nlm.nih.gov

^c^Prot. Score = Protein score, Mowse score ≥ 30

^d^PS = Peptide score

^e^No. P/%C = Number of peptide/% coverage

^f^Name of the gene that codes for the protein sequences, available at http://www.uniprot.org/uploadlists

^g^na, not available

### Differential protein expression in salivary glands between susceptible and resistant strains

**Fig. 3:**
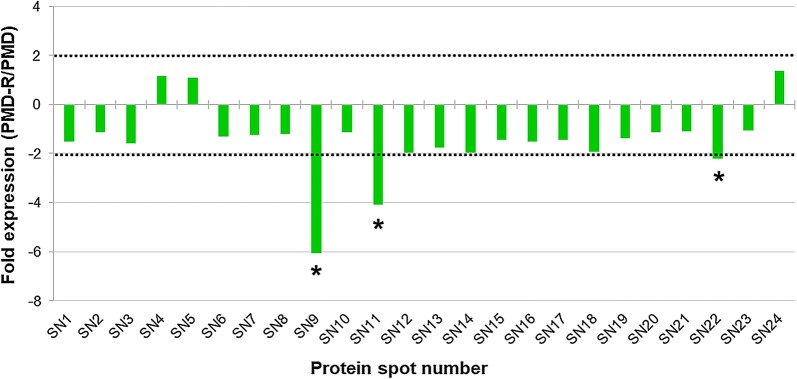
Differential protein expression in salivary glands between PMD and PMD-R strains. Differences in protein expression are represented as expression ratio or fold expression (PMD-R/PMD). Horizontal dot lines indicate the 2-fold difference in expression level in either direction (2 for a higher expression in the PMD-R strain and − 2 for a lower expression in the PMD-R strain). Proteins showing significantly different more than or less than 2-fold expression (Student’s t-test, *P* ≤ 0.05) are indicated by an asterisk (*). The ANV of each major protein and their fold expression values are shown in Additional file 3: Table S2

**Fig. 4:**
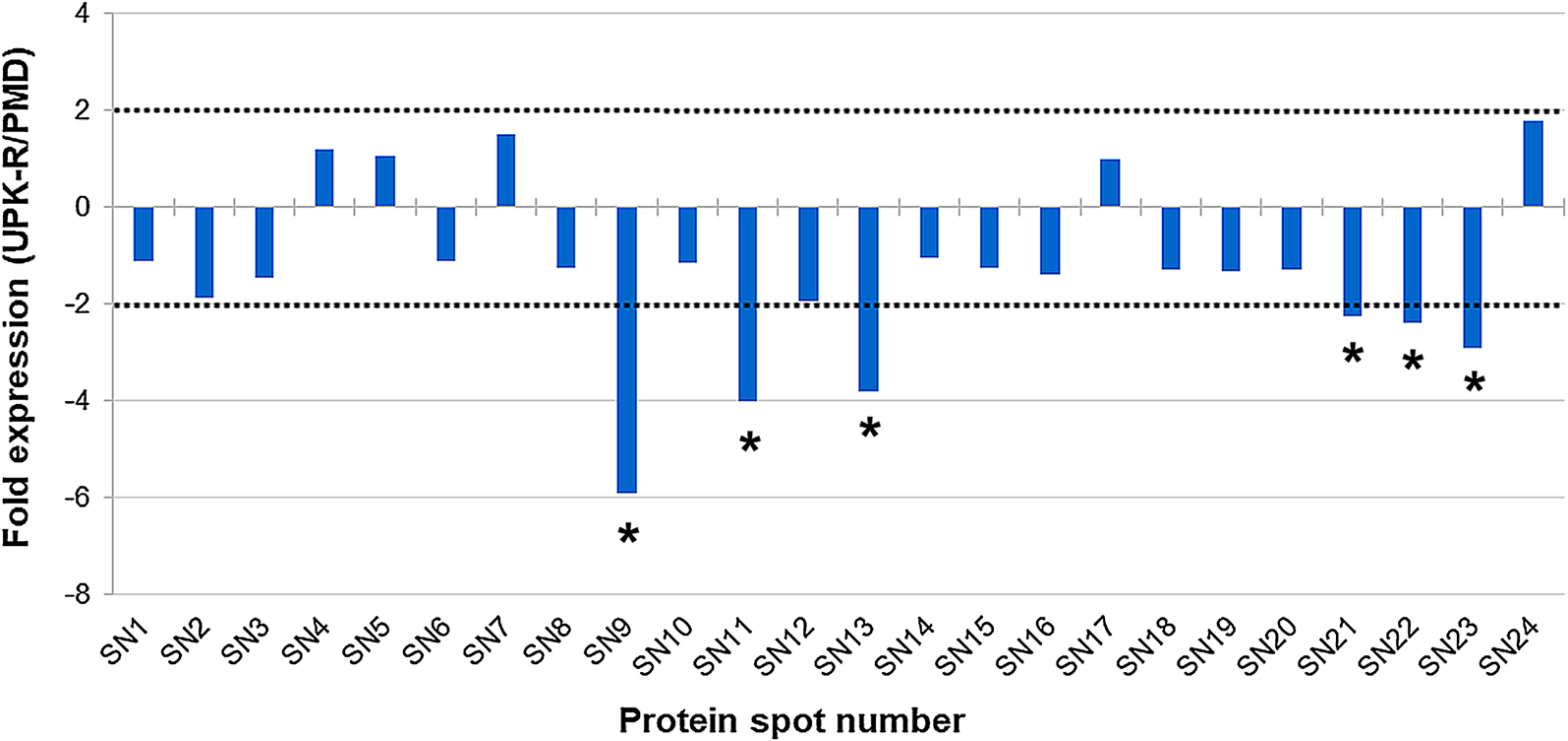
Differential protein expression in salivary glands between PMD and UPK-R strains. Differences in protein expression are represented as expression ratio or fold expression (UPK-R /PMD). Horizontal dot lines indicate the 2-fold difference in expression level in either direction (2 for a higher expression in the UPK-R strain and − 2 for a lower expression in the UPK-R strain). Proteins showing significantly different more than or less than 2-fold expression (Student’s t-test, *P* ≤ 0.05) are indicated by an asterisk (*). The ANV of each major protein and their fold expression values are shown in Additional file 4: Table S3

**Fig. 5:**
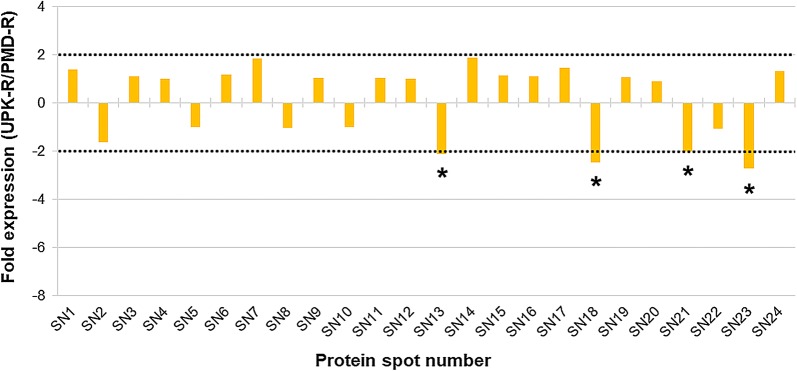
Differential protein expression in salivary glands between PMD-R and UPK-R strains. Differences in protein expression are represented as expression ratio or fold expression (UPK-R /PMD-R UPK-R). Horizontal dot lines indicate the 2-fold difference in expression level in either direction (2 for a higher expression in the UPK-R strain and -2 for a lower expression in the UPK-R strain). Proteins showing significantly different more than or less than 2-fold expression (Student’s t-test, *P* ≤ 0.05) are indicated by an asterisk (*). The ANV of each major protein and their fold expression values are shown in Additional file 5: Table S4

Quantification of the ANV of each major protein of the PMD, PMD-R and UPK-R strains was carried out. The fold expression values of the major proteins were calculated and compared between PMD and PMD-R (Fig. 3 and Additional file 3: Table S2), PMD and UPK-R (Fig. 4 and Additional file 4: Table S3), and PMD-R and UPK-R (Fig. 5 and Additional file 5: Table S4). For PMD-R compared to PMD, three downregulated proteins were identified, namely salivary anti-FXa serpin (SN9), angiopoietin-like protein (SN11) and short form D7Cclu23 salivary protein (SN22) (Fig. 3). For UKP-R compared to PMD, six downregulated proteins were identified, namely salivary anti-FXa serpin (SN9), angiopoietin-like protein (SN11), putative 34 kDa secreted protein (SN13), 30 kDa salivary gland allergen variant 3 (SN21), short form D7Cclu23 salivary protein (SN22) and putative C-type lectin (SN23) (Fig. 4). For PMD-R compared to PMD, four downregulated proteins were identified, namely putative 34 kDa secreted protein (SN13), D7 protein (SN18), 30 kDa salivary gland allergen variant 3 (SN21), and putative C-type lectin (SN23) (Fig. 5).

### Network analysis of identified salivary gland proteins of *Ae. aegypti* PMD, PMD-R and UPK-R strains by STITCH database 5.0

**Fig. 6:**
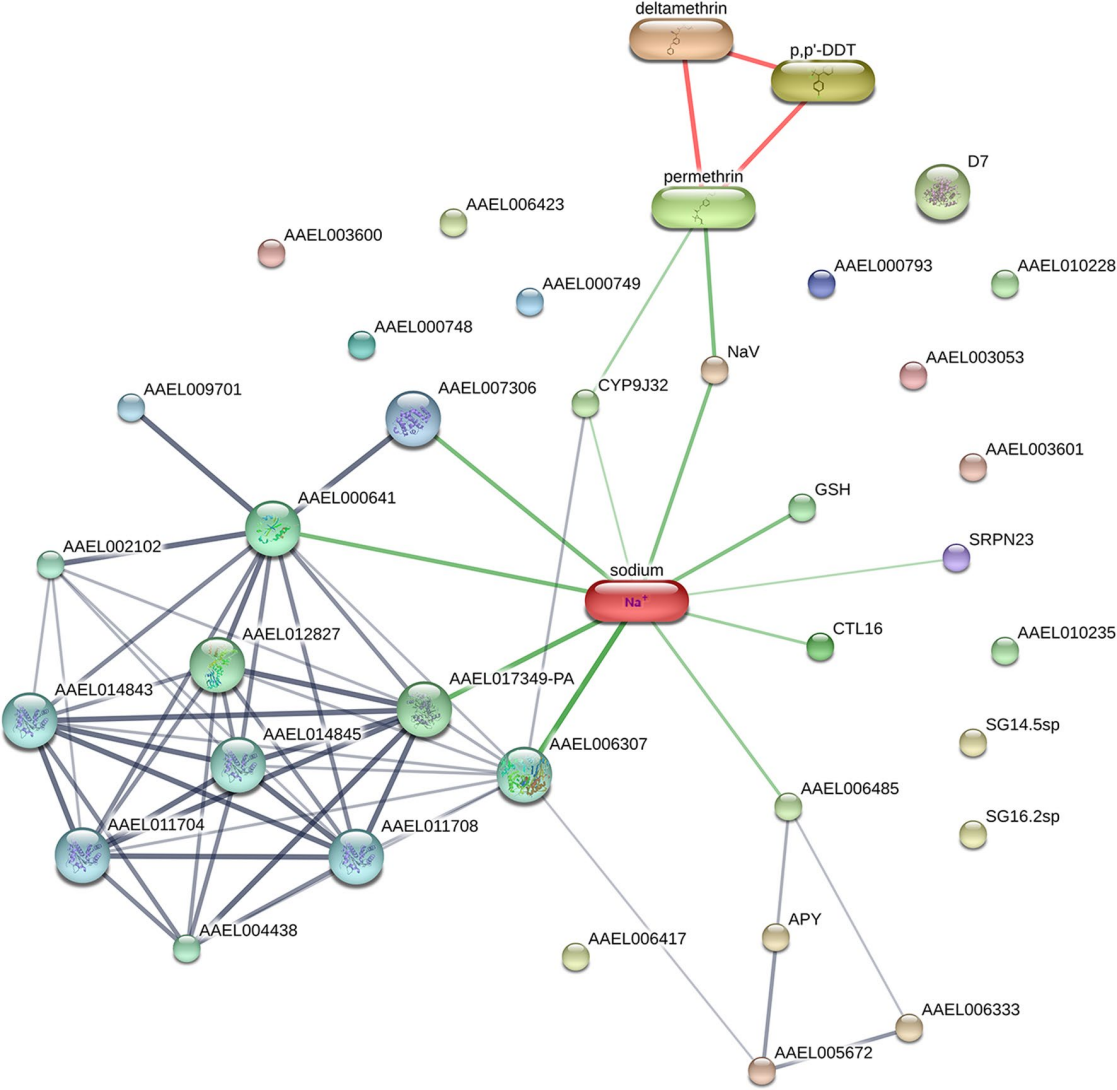
Representative functional associations of salivary gland proteins of adult female *Ae. aegypti* between PMD, PMD-R, and UPK-R strains with other proteins, chemicals and insecticide using the STITCH database 5.0. The predicted functional interaction networks are shown in the network view where the stronger associations are represented by thicker lines. Protein-protein interactions are shown in grey, chemical-protein interactions in green and interactions between chemicals in red. Gene names corresponding to the proteins are described in Table 2

The STITCH database 5.0 was used to analyze potential functional associations of the identified *Ae. aegypti* salivary gland proteins with other proteins, chemicals and insecticides. In this study, the STITCH network of identified protein-insecticide chemical interaction was presented as a network view. Based on this analysis, the interaction of the 24 major proteins of the three strains was similar. The network view showed that eight major proteins, AAEL017349-PA (SN1), apyrase (SN4 and 5), AAEL00633/5’-nucleotidase (SN6), AAEL005672-PA/putative adenosine deaminase (SN7), AAEL000641-PA (SN8), salivary anti-FXa serpin (SN9), putative purine hydrolase (SN10) and putative C-type lectin/AAEL000533-PA (SN23) were found to possibly interact with other proteins (Fig. 6). The confidence scores of interaction between the eight major proteins and other proteins and chemicals are shown in Additional file 6: Table S5.

**Fig. 7:**
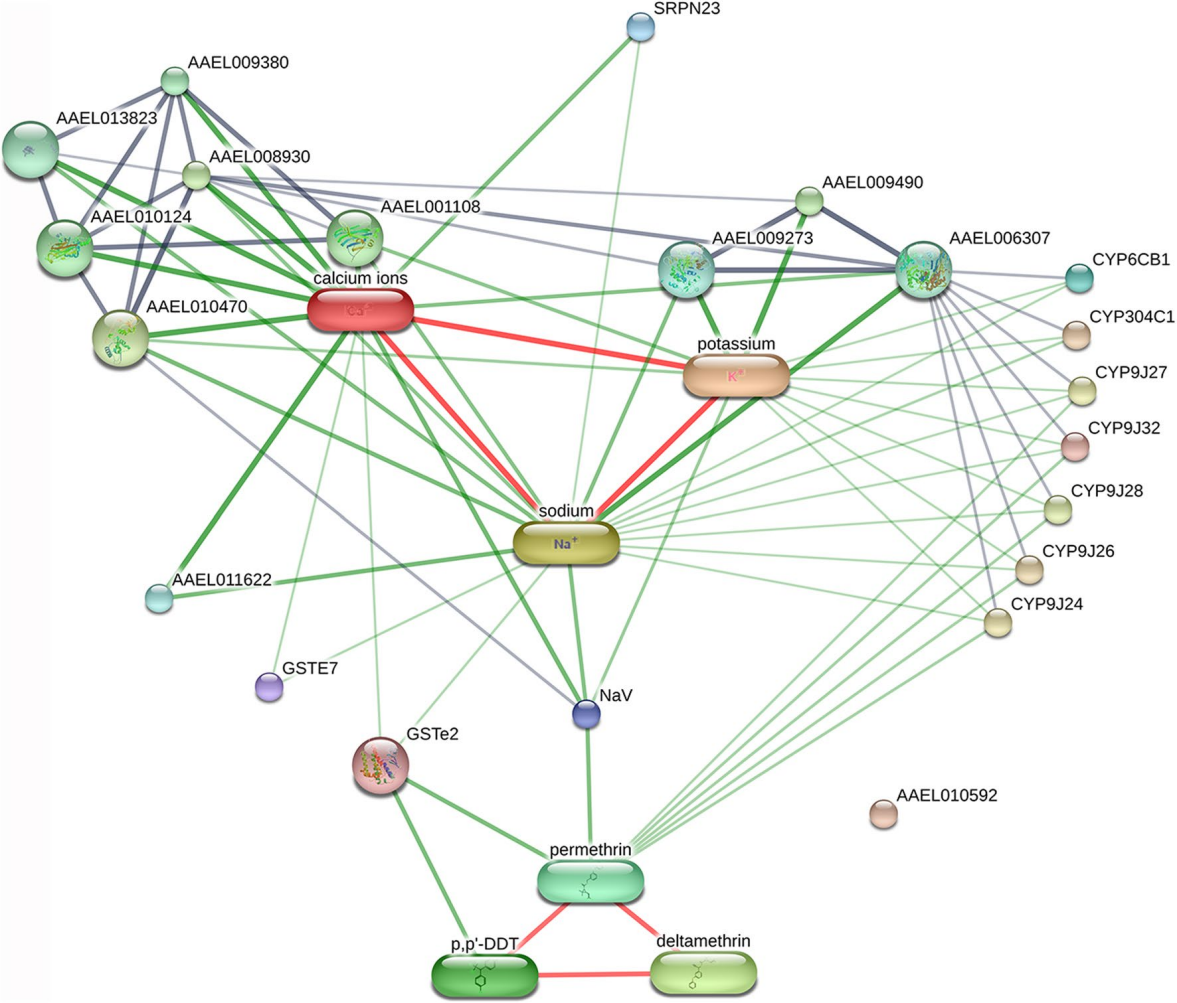
Representative functional association of the SRPN23 with pyrethroid insecticide using the STITCH database 5.0. The predicted functional interaction networks are shown in the network view where the stronger associations are represented by thicker lines. Protein-protein interactions are shown in grey, chemical-protein interactions in green and interactions between chemicals in red. Gene names corresponding to the protein are described in Table 2

Although A2 and A4 proteins had different accession numbers in the NCBI protein database, they had the same gene name (SRPN23) in UniProtKB. Therefore, both A2 and A4 are likely to be the same protein, SRPN23. The network view of the SRPN23 showed that the protein was found interact with sodium and calcium ions (Fig. 7). The confidence scores of interaction between the SRPN23 and sodium and calcium ions are shown in Additional file 7: Table S6.

## Discussion

The present results revealed variation in protein expression profiles and abundance of specific proteins of *Ae. aegypti* salivary glands between insecticide-susceptible and resistant strains. In addition, we were able to identify the specific protein involved. Proteins identified from *Ae. aegypti* salivary glands in this study have been shown to be associated with the facilitation of blood-feeding [21–26]. These proteins include apyrase/5’-nucleotidase (SN4, 5 and 6), putative adenosine deaminase (SN7), salivary anti-FXa serpin (SN9), D7 protein family (SN15, 16, 17, 18, 22 and 24) and 30 kDa salivary gland allergen variant 3 (SN21). Apyrase hydrolyzes ATP and ADP to adenosine by inhibiting ADP-dependent platelet aggregation [23]. Ribeiro et al. [27] proposed that adenosine deaminase activity may help blood-feeding in *Ae. aegypti* by producing inosine and by removing adenosine. Salivary anti-FXa serpin has anti-coagulant properties that inhibits coagulation factor Xa [25]. D7 protein family in *Ae. aegypti* mosquitoes exists in two forms, the long form (~37 kDa) and the short form (~16–18 kDa). The long form D7 salivary proteins function as scavengers of biogenic amines [25]. The short form D7 salivary proteins bind to biogenic amines such as epinephrine, histamine and serotonin, thereby inhibiting platelet aggregation, vasoconstriction and inflammation [22]. Aegyptin is a platelet inhibitor identified in *Ae. aegypti.* It binds to collagens, thereby preventing collagen interaction with Von Willebrand factor, integrin α2β1 and glycoprotein VI [26]. A protein involved in heat-shock response, heat-shock cognate 70 (SN2), was also identified. Zhoa et al. [28] demonstrated that expression level of heat-shock cognate 70 mRNA of female *Ae. aegypti* increases (five- to six-fold) after 42 °C treatment for one hour. Studies by Wasinpiyamongkol et al. [29] and Oktarianti et al. [30] demonstrated that heat-shock cognate 70 (SN2), apyrase/5’-nucleotidase (SN4, 5 and 6), putative adenosine deaminase (SN7), salivary anti-FXa serpin (SN9), angiopoietin-like protein variant, partial (SN11), putative 34 kDa family secreted salivary protein (SN14) and D7 protein family (SN15, 16, 17 and 18) are immunogens.

Differentially expressed protein profiles of the salivary gland proteins of *Ae. aegypti* PMD, PMD-R and UPK-R strains were examined based on the annotated spots in the 2-DE gels and the fold expression values. The results showed that five additional major protein spots (A1-A5) were differentially expressed in the *Ae. aegypti* sialome between the susceptible and resistant strains. A short D7 protein (A1) was specific to the PMD strain whereas a salivary anti-FXa serpin (A2) and A3 were only detected in both the PMD-R and UPK-R strains. A salivary serpin (A4) and A5 were found only in the UPK-R strain. As the A3 and A5 spots were found only in the resistant strains but could not be identified, their DNA and amino acid sequence information is required for further study on their function.

Based on the fold expression values of the major salivary gland proteins, three downregulated proteins (> 2 fold change in expression) were identified in the PMD-R, compared to the PMD including salivary anti-FXa serpin (SN9), angiopoietin-like protein (SN11) and short form D7Cclu23 salivary protein (SN22). Comparison of differentially expressed protein profiles between the PMD and UPK-R strains showed six downregulated proteins in UPK-R compared to PMD including salivary anti-FXa serpin (SN9), angiopoietin-like protein (SN11), putative 34 kDa secreted protein (SN13), 30 kDa salivary gland allergen variant 3 (SN21), short form D7Cclu23 salivary protein (SN22) and putative C-type lectin (SN23). Comparison between the PMD-R and UPK-R strains revealed four downregulated proteins in the UPK-R strain, compared to the PMD-R strain: putative 34 kDa secreted protein (SN13), D7 protein (SN18), 30 kDa salivary gland allergen variant 3 (SN21), and putative C-type lectin (SN23). The results indicated that the pyrethroid resistant strains expressed lower amounts of the differentially expressed major salivary gland proteins than the PMD strain. Furthermore, the UPK-R strain had fold expression values of the differentially expressed proteins that were lower than the PMD-R strain, suggesting that pyrethroids might induce change or alteration of salivary gland proteins and/or their expression in the resistant mosquitoes. Since the change in expression of salivary proteins may impact on the overall fitness of the resistant mosquitoes, these results might help explain blood-feeding and other fitness problems in *Ae. aegypti* female populations with high insecticide resistance levels [31–33].

Djegbe et al. [17] demonstrated four salivary proteins differentially expressed in susceptible (SLAB) and resistant (SR) strains of *Cx. quinquefasciatus* including three metabolic enzymes (endoplasmin, triosephosphate isomerase and heat-shock protein 83) and D7 long form. In susceptible and *ace-1*^*R*^ resistant strains of *An. gambiae* mosquitoes, five proteins regulated in the resistant AceRKis salivary gland extracts have been identified. Two of them, Saglin and TRIO have shown high differences between the susceptible and *ace-1*^*R*^ resistant strains. Both Saglin and TRIO are involved in protection against oxidation, blood-feeding process and pathogen invasion [18]. Vijay et al. [19] reported differentially expressed proteins and enzymes in salivary glands of deltamethrin/DDT resistant strains of *An. stephensi* that may have an impact on insecticide resistance and xenobiotic detoxification, such as short chain dehydrogenase reductase, cytochrome 450 and phosphodiesterase.

A search within UniProtKB (http://www.uniprot.org/uploadlists/) revealed that peptides produced from spots SN9, A2 and A4 all had identity to SRPN23. Given that these spots were found to have variable pIs, it is likely that these spots represent post-translationally modified isoforms of SRPN23. Interestingly, two additional spots, A2 and A4 (SRPN23 isoforms), were expressed only in the PMD-R and UPK-R strains, suggesting that pyrethroids might induce alteration of serpin isoforms in the salivary glands of the resistant mosquitoes by an effect of post-translation modifications (PTM). Insecticide resistance could probably be associated with specific isoform of SRPN23 protein rather than changes in expression of the protein. The potential functional associations of the identified *Ae. aegypti* salivary gland proteins with other proteins, chemicals and insecticides were analyzed by STITCH database 5.0. The results showed that SRPN23 interacted with sodium and calcium ions. These results could warrant investigation of a role of SRPN23 in insecticide resistance because both pyrethroid resistant strains had additional SRPN23 isoforms. Furthermore, testing for the effect of exposure to insecticides in the three strains of *Ae. aegypti* mosquitoes on blood-feeding behavior and metabolic mechanisms should be performed in the future.

Although salivary gland proteins of *Ae. aegypti* mosquito have been studied by several research groups, for example by Valenzuela et al. [21], Ribeiro et al. [22] and Wasinpiyamongkol et al. [29, 34], the status of insecticide resistance of the mosquitoes used in their studies, i.e. *Ae. aegypti* Liverpool/black eye or Bangkok strains, is unknown. In this study, results revealed that the salivary gland proteins of the PMD, PMD-R and UPK-R strains were differentially expressed. Therefore, comparative analyses of the expression of salivary gland proteins of different strains of *Ae. aegypti*, especially the Liverpool/black eye and Rockefeller, 1016 Ile/Ile kdr homozygous strains [31], should be carried out to investigate the expression of salivary proteins due to insecticide resistance.

In summary, to our knowledge this study reports for the first time the proteins expressed differentially in the salivary glands of insecticide-resistant *Ae. aegypti* mosquitoes between pyrethroid-susceptible (PMD) and resistant (PMD-R and UPK-R) strains. Salivary anti-FXa serpin, angiopoietin-like protein and short form D7Cclu23 salivary protein were downregulated in PMD-R when compared to PMD. Six downregulated proteins were detected in UPK-R when compared to PMD, including salivary anti-FXa serpin, angiopoietin-like protein, putative 34 kDa secreted protein, 30 kDa salivary gland allergen variant 3, short form D7Cclu23 salivary protein and putative C-type lectin. Four downregulated proteins were found in the UPK-R when compared to PMD-R, including putative 34 kDa secreted protein, D7 protein, 30 kDa salivary gland allergen variant 3, and putative C-type lectin. Network analysis by STITCH database 5.0 showed that the SRPN23 interacted with sodium and calcium ions.

## Conclusions

This study provides new information on differentially expressed salivary gland proteins in pyrethroid-resistant *Ae. aegypti* mosquitoes. The findings emphasize the requirement for further studies regarding roles of these salivary proteins in viral infection, development and transmission in the resistant strains that might be useful for the development of control strategies for virus transmission by mosquitoes.

## Methods

### Mosquito strains and rearing

Colonies of *Ae. aegypti*, PMD (susceptible to pyrethroids but resistant to DDT), PMD-R (resistant to DDT and permethrin) and UPK-R (resistant to DDT, permethrin and deltamethrin) strains were successfully maintained in an insectary of the Department of Parasitology, Faculty of Medicine, Chiang Mai University, Thailand and utilized in this study. *Ae. aegypti* PMD and PMD-R strains were established from field-caught mosquitoes from Ban Pang Mai Daeng, Mae Taeng District, Chiang Mai Province and maintained in an insectary at the Department of Parasitology, Faculty of Medicine, Chiang Mai University, Thailand since 1997 [5]. The UPK-R strain was established from wild-caught mosquitoes from Wat Upakhut, Chiang Mai city and maintained in an insectary at the Department of Parasitology, Faculty of Medicine, Chiang Mai University, Thailand since 2006 [14]. The permethrin resistance level of UPK-R and PMD-R strains, as determined by larval bioassays, is higher than the PMD strain by 325-fold and 25-fold, respectively ([10, 11], P. Somboon unpublished data). The deltamethrin resistance level of the UPK-R and PMD-R strains, as determined by larval bioassays, is higher than the susceptible PMD strain by 53-fold and 13-fold, respectively [14]. For each strain, a total of 300–400 eggs were placed into a 25 × 35 × 6 cm plastic tray filled with 3 l of distilled water and allowed to hatch. After hatching, the larvae were fed on finely ground dog food and the water was changed three times per week. Pupae were collected and transferred to plastic cups containing distilled water and then placed into a 30 × 30 × 30 cm mosquito cage. After emergence, the mosquitoes were maintained in the insectary at 27 ± 2 °C with a relative humidity of 70 ± 10% and a light-dark photocycle of 12:12 h. The mosquitoes were provided with 10% sucrose solution. They were allowed to feed on adult albino rats, *Rattus norvegicus*, to produce eggs. To avoid contamination of the mosquito strains, each strain was separately maintained in a different room of our insectary in the Department of Parasitology. Sugar-fed females aged five to seven days post-emergence were used in this study.

### Insecticide susceptibility test

The mosquitoes from PMD-R and UPK-R colonies had been exposed regularly to the standard WHO permethrin (0.75%) and deltamethrin (0.05%) papers, respectively, to maintain their insecticide resistant status. Insecticide susceptibility tests were performed on all mosquito strains according to the protocol described by WHO [20]. Briefly, 25 one-day-old unfed female mosquitoes were exposed to insecticide impregnated paper in an exposure tube for 1 h and transferred to the holding tube. The mortality rate was counted after 24 h post-exposure. Control mosquitoes were exposed to paper without insecticide for 1 h and the mortality rate was counted after 24 h post-exposure. Two replicates of the control group and four replicates of each test were performed each time. The mosquitoes that were able to fly and rest on the paper were recorded as survived or resistant. Mortality of 5–20% was accepted in the control groups and Abbott’s formula was used to correct the mortality rate in the test groups [35]. If the mortality in the control group was over 20%, all tests were discarded.

### Salivary gland dissection and protein quantification

The salivary glands of females of the three strains were dissected using the method described by Jariyapan et al. [36]. Mosquitoes of each strain were anaesthetized on ice before dissection. Salivary glands were dissected in phosphate-buffered saline (PBS), washed with PBS on a new slide to clean them from other contaminated tissues, and collected in a microcentrifuge tube at -80 °C until use. The protein content was determined using a Micro BCA Protein Assay Kit (Pierce, Rockford, IL, USA).

### Sds-page

SDS-gel loading buffer (50 mM Tris-HCl, pH 6.8, 100 mM DTT, 2% SDS, 0.1% bromophenol blue, 10% glycerol), 1:2 (v/v), was added into each salivary gland sample of the three strains. The samples were heated for 5 min before loading on 15% SDS polyacrylamide gels. Molecular weight markers (Bio-Rad, Hercules, California, USA) were applied in each gel [37, 38].

### Two-dimensional gel electrophoresis

Two-dimensional gel electrophoresis was performed using a 2D system from GE Healthcare (Buckinghamshire, UK) as described in our previous studies by Sor-suwan et al. [37] and Phattanawiboon et al. [38]. Briefly, 60 pairs of female salivary glands (≈80 µg) were used as a sample for 2-DE analysis. 2-DE samples were collected from different cohorts of each mosquito strain. Samples from each strain were subjected to triplicate runs. A 2-D Clean-Up kit (GE Healthcare) was used to desalt the salivary gland samples. For the first dimension, each sample was diluted in a 125 µl rehydration solution (8 M urea, 50 mM DTT, 0.2% 3/10 Bio-lyte Ampholyte, 4% CHAPS, 0.002% bromophenol blue). This solution was applied onto an IPG strip (7 cm, pI 3–10, GE Healthcare). The IPG strip was submitted to isoelectric focusing on an Ettan IPGphor III (GE Healthcare) which was operated as described in Phattanawiboon et al. [38]. After that, the focused IPG strip was incubated in 10 ml SDS equilibration buffer (6 M urea, 0.05 M Tris, pH 8.8, 2% SDS, 30% glycerol, 0.002% bromophenol blue) containing 100 mg DTT for 15 min and then in 5 ml SDS equilibration buffer containing 125 mg iodoacetamide for 15 min. The equilibrated strip was applied to the surface of vertical 15% SDS polyacrylamide gels, sealed with 0.1% agarose. The Mini-PROTEAN Tetra Electrophoresis System (Bio-Rad) was used to separate proteins in the second dimension. Molecular weight markers (broad range from Bio-Rad) were loaded on each gel.

### Coomassie Brilliant Blue (CBB) staining and gel image analysis

After electrophoresis, the gels were stained with CBB and spots in each gel and their expression volume were analyzed as described in our previous studies by Phattanawiboon et al. [38].

### Statistical analysis

Image Master 2D Platinum 7.0 software (GE Healthcare) was used to measure the density of all protein spots and quantify the ANV for each protein spot. Statistical analysis (Student’s t-test, *P* ≤ 0.05) was performed using SPSS version 22.0 software (SPSS, Chicago, IL, USA) with a cut-off of 2-fold up- or downregulated to compare the ANV of each protein spot between two strains, i.e. PMD and PMD-R, PMD and UPK-R, and PMD-R and UPK-R strains.

### In-gel digestion

Protein spots of interest were cut from the 2-DE gels using sterile surgical blades ensuring that technique was aseptic. Each spot sample was placed separately in a sterile microcentrifuge tube. The samples were subjected to in-gel digestion using methods described in our previous studies by Sor-suwan et al. [37] and Phattanawiboon et al. [38]. The gel plugs were dehydrated with 100% acetonitrile (ACN), reduced with 10 mM DTT in 10 mM ammonium bicarbonate at room temperature for 1 h, and alkylated at room temperature for 1 h in the dark in the presence of 100 mM iodoacetamide (IAA) in 10 mM ammonium bicarbonate. After alkylation, the gel pieces were dehydrated twice with 100% ACN for 5 min. The gels were digested in 10 µl of trypsin solution (10 ng/µl trypsin in 50% ACN/10 mM ammonium bicarbonate at room temperature for 20 min, and then 20 µl of 30% ACN) were added. After that the gel samples were incubated at 37 °C for a few hours or overnight. Products of digested proteins from the gels were extracted by adding 40 µl of 50% ACN in 0.1% formic acid (FA) and shaking for 10 min at room temperature. Extracted peptides were collected in a new tube. The pool-extracted peptides were dried by a vacuum centrifuge and kept at -80 °C for further analysis.

### NanoLC-MS analysis and protein identification

NanoLC-MS analysis of the pool-extracted peptides was performed as a method described in Phattanawiboon et al. [38]. The pool-extracted samples were injected into an Ultimate 3000 LC System (Dionex, Sunnyvale, California, USA) coupled to an ESI-Ion Trap MS (HCT Ultra PTM Discovery System, Bruker, Germany) with electrospray at a flow rate of 300 nl/min to a nanocolumn (Acclaim PepMap 100 C18, 3 µm, 100 A, 75 µm id × 150 mm). A solvent gradient (solvent A: 0.1% formic acid in water; solvent B: 80% 0.1% formic acid in 80% acetonitrile) was run for 40 min. Mascot from Matrix Science Ltd. (London, UK) was used to search all of the tandem mass spectra [39]. The resulting sequence data was searched against the National Center for Biotechnology Information nonredundant (NCBInr) protein database. The parameters for searching were as follows: enzyme of specificity strict trypsin; three missed cleavages; fixed modification (carbamidomethyl); variable modification (Methionine oxidation); peptide tolerance, variable from 50 ppm to 100 ppm; fragment mass tolerance of ± 0.5 Da; peptide change of 1+, 2+, and 3+; and monoisotopic. Proteins identified with a statistically significant Mowse score (≥ 30) was reported. Gene ontology analysis was performed using UniProtKB (http://www.uniprot.org/uploadlists/) for molecular function, biological processes and cellular component. The mapping of protein-chemical interactions was analyzed according to STITCH 5.0 database (http://stitch.embl.de/) and KEGG (Kyoto Encyclopedia of Genes and Genomes) PATHWAY data.

## Additional files

**Additional file 1: Figure S1.** Three independent biological replicated 2-DE gel images of each strain.

**Additional file 2: Table S1.** The mass list (list of peptides) found for each major spot analyzed on the three mosquito strains.

**Additional file 3: Table S2.** Differential protein expression in salivary glands between PMD and PMD-R strains.

**Additional file 4: Table S3.** Differential protein expression in salivary glands between PMD and UPK-R strains.

**Additional file 5: Table S4.** Differential protein expression in salivary glands between PMD-R and UPK-R strains.

**Additional file 6: Table S5.** The confidence scores of interaction between the eight major proteins and other proteins and chemicals.

**Additional file 7: Table S6.** The confidence scores of interaction between the SRPN23 and sodium and calcium ions.

## Abbreviations

ACN: acetonitrile
ANV: average normalized volume
BSA: bovine serum albumin
CBB: Coomassie Brilliant Blue
CHAPS: 3-[(3-cholamidopropyl)dimethyl-ammonio]-1-propanesulfonate
CHIKV: chikungunya virus
CRISPR-Cas9: clustered regularly interspaced short palindromic repeats-associated protein 9
DDT: dichlorodiphenyltrichloroethane
DENV: dengue virus
DHF: dengue hemorrhagic fever
DTT: dithiothreitol
FA: formic acid
IAA: iodoacetamide
IEF: isoelectric focusing
IPG: immobilized pH gradient
kDa: kilodalton
kdr: knockdown resistance
KEGG: Kyoto Encyclopedia of Genes and Genomes
MW: molecular weight
NanoLC-MS: nano liquid chromatography-mass spectrometry
Na_2_SO_4_: sodium sulfate
NCBInr: National Center for Biotechnology Information nonredundant
PBS: phosphate-buffered saline
pI: isoelectric points
PMD: Pang Mai Dang susceptible strain
PMD-R: Pang Mai Dang resistant strain
PTM: post-translation modifications
RNAi: RNA interference
SDS: sodium dodecyl sulfate
SDS-PAGE: sodium dodecyl sulphate polyacrylamide gel electrophoresis
SN: spot number
STITCH: Search Tool for Interacting Chemicals
UPK-R: Upakut resistant strain
WHO: World Health Organization
2-DE: two-dimensional gel electrophoresis
