# Supplementary figures and images for: Protein expression in female salivary glands of pyrethroid-susceptible and resistant strains of Aedes aegypti mosquitoes
Source: Parasit Vectors. 2019 Mar 14;12:111. doi: 10.1186/s13071-019-3374-2 (PMC6419353; doi:10.1186/s13071-019-3374-2)

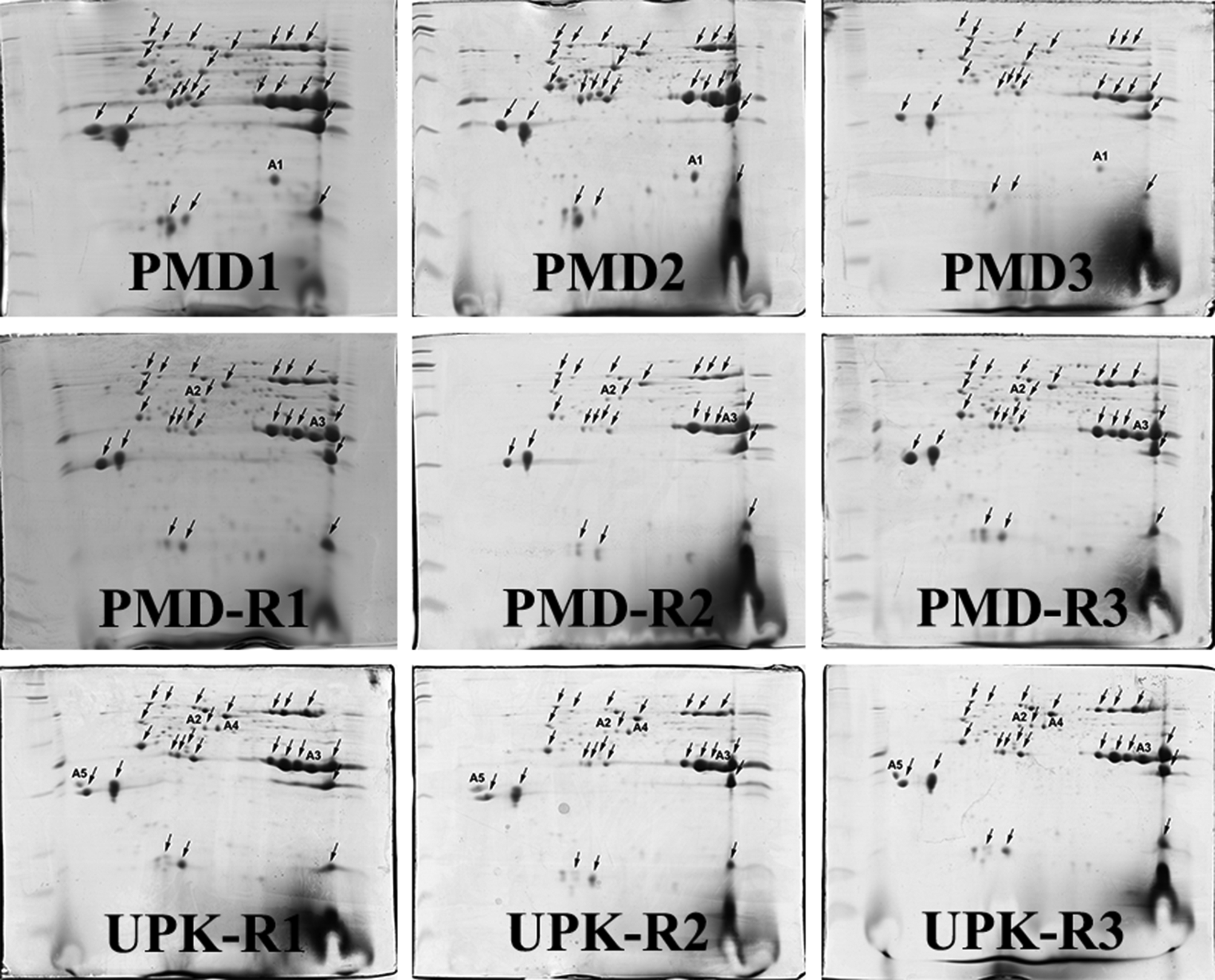

Supplement: Supplementary file 1 — Additional file 1: Figure S1. Three independent biological replicated 2-DE gel images of each strain. [file 13071_2019_3374_MOESM1_ESM.tif]
