# Supplementary material for: Protein expression in female salivary glands of pyrethroid-susceptible and resistant strains of Aedes aegypti mosquitoes
Source: Parasit Vectors. 2019 Mar 14;12:111. doi: 10.1186/s13071-019-3374-2 (PMC6419353; doi:10.1186/s13071-019-3374-2)
Supplement: Supplementary file 3 — Additional file 3: Table S2. Differential protein expression in salivary glands between PMD and PMD-R strains. [file 13071_2019_3374_MOESM3_ESM.docx]

**Additional file 3: Table S2.** Differential protein expression in salivary glands between PMD and PMD-R strains

| SN^a^ | MW/pI^b^ | ANV±SD^c^ | | X-Fold Average |
| --- | --- | --- | --- | --- |
|  |  | PMD | PMD-R |  |
| Up-regulated proteins | | | | |
| 4 | 69/8.5 | 0.59±0.01 | 0.75±0.00 | 1.17 |
| 5 | 68/8.9 | 1.24±0.02 | 1.35±0.04 | 1.09 |
| 24 | 15/5.9 | 0.25±0.00 | 0.43±0.00 | 1.37 |
| Down-regulated proteins | | | | |
| 1 | 75/5.3 | 0.33±0.01 | 0.22±0.00 | 1.52 |
| 2 | 73/5.6 | 0.22±0.01 | 0.19±0.00 | 1.15 |
| 3 | 72/6.2 | 0.34±0.01 | 0.21±0.01 | 1.59 |
| 6 | 68/9.5 | 2.56±0.03 | 1.98±0.03 | 1.29 |
| 7 | 66/7.2 | 0.77±0.03 | 0.63±0.00 | 1.23 |
| 8 | 62/5.1 | 0.49±0.02 | 0.41±0.00 | 1.19 |
| 9 | 59/6.6 | 0.81±0.01 | 0.13±0.03 | 6.06^d^ |
| 10 | 49/5.1 | 0.86±0.03 | 0.77±0.05 | 1.13 |
| 11 | 48/6.0 | 0.58±0.01 | 0.12±0.02 | 4.07^d^ |
| 12 | 45/5.8 | 0.56±0.01 | 0.40±0.00 | 1.95 |
| 13 | 46/5.7 | 0.73±0.00 | 0.47±0.00 | 1.77 |
| 14 | 43/6.2 | 0.90±0.01 | 0.46±0.00 | 1.96 |
| 15 | 40/9.5 | 9.70±0.03 | 6.66±0.00 | 1.46 |
| 16 | 39/9.5 | 7.23±0.05 | 4.72±0.00 | 1.53 |
| 17 | 40/8.9 | 7.10±0.01 | 4.93±0.00 | 1.44 |
| 18 | 42/8.5 | 3.35±0.01 | 7.68±0.00 | 1.94 |
| 19 | 33/9.5 | 6.32±0.02 | 4.55±0.00 | 1.39 |
| 20 | 31/4.1 | 5.04±0.02 | 4.03±0.00 | 1.13 |
| 21 | 33/4.6 | 4.17±0.00 | 3.56±0.09 | 1.11 |
| 22 | 15/9.5 | 5.82±0.01 | 2.65±0.00 | 2.20^d^ |
| 23 | 15/5.6 | 0.43±0.00 | 0.40±0.03 | 1.06 |

^a^Spot number refers to those shown in Fig. 2

^b^Observed molecular mass and isoelectric point

^c^Average Normalized Volume ± Standard Deviation

^d^Student’s t-test, P≤0.05 with a cut-off of 2-fold
