# Supplementary material for: Protein expression in female salivary glands of pyrethroid-susceptible and resistant strains of Aedes aegypti mosquitoes
Source: Parasit Vectors. 2019 Mar 14;12:111. doi: 10.1186/s13071-019-3374-2 (PMC6419353; doi:10.1186/s13071-019-3374-2)
Supplement: Supplementary file 5 — Additional file 5: Table S4. Differential protein expression in salivary glands between PMD-R and UPK-R strains. [file 13071_2019_3374_MOESM5_ESM.docx]

**Additional file 5: Table S4.** Differential protein expression in salivary glands between PMD-R and UPK-R strains

| SN^a^ | MW/pI^b^ | ANV±SD^c^ | | X-Fold Average |
| --- | --- | --- | --- | --- |
|  |  | PMD-R | UPK-R |  |
| Up-regulated proteins | | | | |
| 1 | 75/5.3 | 0.22±0.00 | 0.29±0.05 | 1.36 |
| 3 | 72/6.2 | 0.21±0.01 | 0.23±0.03 | 1.09 |
| 4 | 69/8.5 | 0.75±0.00 | 1.05±0.02 | 1.00 |
| 6 | 68/9.5 | 1.98±0.03 | 2.32±0.01 | 1.17 |
| 7 | 66/7.2 | 0.63±0.00 | 1.15±0.01 | 1.83 |
| 9 | 59/6.6 | 0.13±0.03 | 0.14±0.03 | 1.02 |
| 11 | 48/6.0 | 0.12±0.02 | 0.12±0.02 | 1.01 |
| 12 | 45/5.8 | 0.40±0.00 | 0.44±0.03 | 1.00 |
| 14 | 43/6.2 | 0.46±0.00 | 0.85±0.02 | 1.85 |
| 15 | 40/9.5 | 6.66±0.00 | 7.62±0.02 | 1.14 |
| 16 | 39/9.5 | 4.72±0.00 | 5.12±0.03 | 1.09 |
| 17 | 40/8.9 | 4.93±0.00 | 7.11±0.01 | 1.44 |
| 19 | 33/9.5 | 4.55±0.00 | 4.83±0.03 | 1.06 |
| 20 | 31/4.1 | 4.034±0.00 | 4.09±0.04 | 0.88 |
| 24 | 15/5.9 | 0.43±0.00 | 0.66±0.03 | 1.31 |
| Down-regulated proteins | | | | |
| 2 | 73/5.6 | 0.19±0.00 | 0.12±0.01 | 1.63 |
| 5 | 68/8.9 | 1.35±0.04 | 1.33±0.01 | 1.02 |
| 8 | 62/5.1 | 0.41±0.00 | 0.40±0.03 | 1.04 |
| 10 | 49/5.1 | 0.77±0.05 | 0.75±0.03 | 1.02 |
| 13 | 46/5.7 | 0.47±0.00 | 0.22±0.00 | 2.14^d^ |
| 18 | 42/8.5 | 7.68±0.00 | 2.62±0.00 | 2.48^d^ |
| 21 | 33/4.6 | 3.56±0.09 | 2.54±0.01 | 2.03^d^ |
| 22 | 15/9.5 | 2.65±0.00 | 2.25±0.02 | 1.08 |
| 23 | 15/5.6 | 0.40±0.03 | 0.15±0.03 | 2.73^d^ |

^a^Spot number refers to those shown in Fig. 2

^b^Observed molecular mass and isoelectric point

^c^Average Normalized Volume ± Standard Deviation

^d^Student’s t-test, P≤0.05 with a cut-off of 2-fold
