# Supplementary material for: Protein expression in female salivary glands of pyrethroid-susceptible and resistant strains of Aedes aegypti mosquitoes
Source: Parasit Vectors. 2019 Mar 14;12:111. doi: 10.1186/s13071-019-3374-2 (PMC6419353; doi:10.1186/s13071-019-3374-2)
Supplement: Supplementary file 6 — Additional file 6: Table S5. The confidence scores of interaction between the eight major proteins and other proteins and chemicals. [file 13071_2019_3374_MOESM6_ESM.docx]

**Additional file 6: Table S5.** The confidence scores of interaction between the eight major proteins and other proteins and chemicals

| **Gene name (description of identified proteins)^a^** | **Gene name or chemical (description of proteins or chemicals)^b^** | **Confidence score^c^** |
| --- | --- | --- |
| AAEL017349-PA (AAEL017349-PA) | AAEL012827 (endoplasmin) | 0.996 |
|  | AAEL004438 (conserved hypothetical protein) | 0.994 |
|  | AAEL011704 (heat shock protein) | 0.987 |
|  | AAEL011708 (heat shock protein) | 0.987 |
|  | AAEL014843 (heat shock protein) | 0.987 |
|  | AAEL014845 (heat shock protein) | 0.987 |
|  | Sodium (sodium ion) | 0.876 |
|  | AAEL000641 (protein disulfide isomerase) | 0.757 |
|  | AAEL006307 (acetyl-coA carboxylase) | 0.537 |
|  | AAEL002102 (conserved hypothetical protein) | 0.520 |
| APY (apyrase precursor/apyrase) | AAEL005672 (adenosine deaminase) | 0.700 |
|  | AAEL006485 (inosine-uridine preferring nucleoside hydrolase) | 0.546 |
| AAEL006333 (salivary apyrase, putative/5'nucleotidase/AAEL006333-PA) | AAEL005672 (adenosine deaminase) | 0.737 |
|  | AAEL006485 (inosine-uridine preferring nucleoside hydrolase) | 0.432 |
| AAEL005672 (adenosine deaminase, putative) | AAEL006333 (salivary apyrase, putative) | 0.737 |
|  | APY (apyrase precursor) | 0.700 |
|  | AAEL006307 (acetyl-coA carboxylase) | 0.416 |
| AAEL000641 (protein disulfide isomerase/AAEL000641-PA) | AAEL002102 (conserved hypothetical protein) | 0.990 |
|  | AAEL007306 (alpha-actinin) | 0.980 |
|  | AAEL009701 (conserved hypothetical protein) | 0.980 |
|  | AAEL012827 (endoplasmin) | 0.952 |
|  | AAEL011704 (heat shock protein) | 0.803 |
|  | AAEL011708 (heat shock protein) | 0.803 |
|  | AAEL014843 (heat shock protein) | 0.803 |
|  | AAEL014845 (heat shock protein) | 0.803 |
|  | Sodium (sodium ion) | 0.772 |
|  | AAEL017349-PA (AAEL017349-PA) | 0.757 |
|  | AAEL006307 (acetyl-coA carboxylase) | 0.489 |
| SRPN23 (salivary anti FXa serpin/salivary serpin) | Sodium (sodium ion) | 0.412 |
| AAEL006485 (inosine-uridine preferring nucleoside hydrolase/putative purine hydrolase) | Sodium (sodium ion) | 0.577 |
|  | APY (apyrase precursor) | 0.546 |
|  | AAEL006333 (salivary apyrase, putative) | 0.432 |
| CTL16 (C-Type lectin (CTL), putative/AAEL000533-PA) | Sodium (sodium ion) | 0.535 |

^a^The eight major proteins identified in this study

^b^Proteins or chemicals that were predicted by STITCH database 5.0 that interacted with the identified proteins

^c^The range of confidence scores by STITCH database 5.0 is 0-1 and 1 is the highest possible confidence
