# Supplementary material for: Protein expression in female salivary glands of pyrethroid-susceptible and resistant strains of Aedes aegypti mosquitoes
Source: Parasit Vectors. 2019 Mar 14;12:111. doi: 10.1186/s13071-019-3374-2 (PMC6419353; doi:10.1186/s13071-019-3374-2)
Supplement: Supplementary file 7 — Additional file 7: Table S6. The confidence scores of interaction between the SRPN23 and sodium and calcium ions. [file 13071_2019_3374_MOESM7_ESM.docx]

**Table S6.** The confidence scores of interaction between the SRPN23 and sodium and calcium ions

| **Gene name (description of identified protein)^a^** | **Chemicals (description of chemicals)^b^** | **Confidence score^c^** |
| --- | --- | --- |
| SRPN23 (salivary anti FXa serpin/salivary serpin) | Sodium (sodium ion) | 0.412 |
|  | Calcium (calcium ion) | 0.731 |

^a^The protein identified in this study

^b^Chemicals that were predicted by STITCH database 5.0 that interacted with the identified protein

^c^The range of confidence scores by STITCH database 5.0 is 0-1 and 1 is the highest possible confidence
